# Flavonoids as Potential Therapeutic Agents Against Gastrointestinal Pathogen Growth and Their Mechanisms of Action: A Comprehensive Review

**DOI:** 10.3390/molecules31132265

**Published:** 2026-06-29

**Authors:** Muhammad Jawad Yousaf Zai, Ian Edwin Cock

**Affiliations:** 1 Biodiversity Research Centre, Dhofar University, Salalah 211, Oman; myousafzai@du.edu.om; 2 Centre of Planetary Health and Food Security, Griffith University, Brisbane, QLD 4111, Australia; 3 School of Environment and Science, Griffith University, Brisbane, QLD 4111, Australia

**Keywords:** flavonoids, gastrointestinal pathogens, antimicrobial therapies, combinational therapies

## Abstract

The increasing prevalence of gastrointestinal infections, along with growing resistance to conventional antibiotics, has driven the search for alternative antibacterial agents including flavonoids, which are widely recognised for their antimicrobial properties. Flavonoids exhibit broad-spectrum antimicrobial activity against both Gram-positive and Gram-negative bacteria through multifactorial mechanisms, disrupting multiple bacterial cellular processes, including energy metabolism, membrane integrity, and protein synthesis. Several studies have highlighted the antimicrobial potential of flavonoids against gastrointestinal pathogens, with some evidence suggesting minimal disruption to beneficial intestinal microflora. Most investigations have primarily focused on in vitro assessments of antibacterial activity, with limited in vivo studies and insufficient therapeutic or clinical evaluation. Consequently, their efficacy in physiological systems and the underlying mechanisms of action remain inadequately understood. Studies should also examine the antimicrobial activity of flavonoids against a wider panel of gastrointestinal pathogens. Also, the panel of flavonoids previously evaluated for their antibacterial activity is relatively narrow and many compounds have been neglected to date. Multiple other flavonoids remain to be evaluated for antimicrobial activity against gastrointestinal pathogens. This review focuses on the antimicrobial activity of different classes of flavonoids against gastrointestinal pathogens. Additionally, we discuss the interaction of selected flavonoids with conventional antibiotics. Furthermore, their mechanisms of action (where known) are also discussed to focus attention on the use of this important class of molecules as antibiotic therapies.

## 1. Introduction

Acute gastroenteritis (AGE) remains a significant cause of mortality and morbidity globally [1]. Bacteria, viruses, toxins, and parasites are among the major pathogenic factors associated with AGE [2]. Most AGE cases are associated with infection by at least one foodborne pathogen [2]. Despite its relatively low mortality rate, the high prevalence of AGE results in considerable morbidity and economic strain on healthcare systems [3,4]. AGE is responsible for an estimated 89.5 million disability adjusted life years (DALYs) lost and 1.45 million deaths worldwide each year [5]. In the United States of America (USA), AGE represents a major cause of disease, with an estimated 179 million cases each year [6]. However, this figure may be substantially underestimated as many cases of AGE resolve without medical intervention and remain unreported. In China, AGE remains a major public health issue, and a population surveillance study carried out in selected provinces from 2010 to 2011 offered an initial evaluation of its burden [7]. Data collected from six provinces showed that the weighted monthly prevalence of AGE was 4.2%, with an incidence rate of 0.56 cases per person per year [8]. Assessment of AGE disease burden is an essential step in evaluating the overall burden of foodborne diseases, a major global public health concern [1]. In high-income countries with low AGE mortality, long-term complications of *Salmonella* and *Campylobacter* infections may account for a higher DALY burden than the acute illness [9,10].

The human gastrointestinal (GI) microbiome comprises a diverse community of microorganisms, including bacteria, archaea, lower and higher eukaryotes, and viruses that reside within the GI tract [11]. These microbial populations play essential roles in nutrient provision and in protecting the host against pathogenic infections [12]. However, opportunistic pathogens can become part of the gut microbiota, particularly in immunocompromised individuals. Such pathogens may include virulent variants of normal intestinal flora, such as *Enterococcus faecium* and *Escherichia coli*, as well as established pathogens, including *Clostridioides difficile* [13]. Owing to its extensive microbial diversity, the GI microbiome can also function as a reservoir for antibiotic resistant bacteria [14]. *Helicobacter pylori* exhibits particularly high levels of antibiotic resistance, with reported resistance rates of 50–80% metronidazole-resistant infections, 30–35% to levofloxacin, and 20–40% to clarithromycin [15]. Comparable resistance has been documented in *Clostridiodes difficile* (51% to cephalosporins and 8.3–100% to clindamycin), *Campylobacter coli* and *Campylobacter jejuni* (approximately 85% resistance to fluoroquinolones), *E. coli* (76.5% to ampicillin), and Gram-negative pathogens responsible for bacterial peritonitis (85% resistance to methicillin and about 40% to third-generation cephalosporins) [15]. Antibiotic resistance arises through multiple mechanisms, including prevention of antibiotic binding to cellular targets, structural modification or degradation of antimicrobial agents and via efflux pumps [16]. A resistance trait may develop through genetic mutations that are passed to daughter cells during division. Additionally, bacteria can acquire resistance genes via horizontal gene transfer (HGT), involving mechanisms such as transformation, transduction, conjugation, and DNA transfer mediated by membrane vesicles. Collectively, these processes facilitate the dissemination of antibiotic resistance genes among bacteria sharing the same environment [16].

Natural products are an essential source of therapeutic agents and provide a structural framework for the development of modern synthetic drugs, including antimicrobial agents [17]. The rapid emergence of plasmid-mediated antibiotic resistance, together with the persistence of diseases insufficiently managed by current therapies, has intensified interest in discovering novel antimicrobial compounds from natural sources. The World Health Organisation (WHO) supports the use of medicinal plants as complementary therapeutic options, particularly where conventional treatments are limited [17]. Consequently, increasing attention has been directed toward identifying plant-derived bioactive compounds, characterising their chemical composition, and evaluating their pharmacological potential, especially for compounds exhibiting lower toxicity than existing drugs. Natural products are now widely investigated for the management of microbial infections, cancer, and inflammatory disorders, largely due to their accessibility and promising therapeutic efficacy [18]. Medicinal plants represent one of the largest reservoirs of bioactive molecules for drug discovery, and the expanding global demand for herbal medicines reflects their growing clinical and commercial importance. Market analysis estimated that the global herbal medicine market increased from USD 29.4 billion in 2017 to approximately USD 39.6 billion by 2022 [19].

Flavonoids are plant secondary metabolites that exhibit a wide range of pharmacological activities, including antimicrobial effects [20,21]. This review article aims to highlight the antimicrobial activity of flavonoids against gastrointestinal pathogens, as well as their mechanisms of action. Synergistic interactions arise when two or more compounds act together to produce a greater therapeutic effect than their individual effects combined. This review will also highlight synergistic interactions between flavonoids and conventional antibiotics.

## 2. Classes of Flavonoids

**Figure 1:**
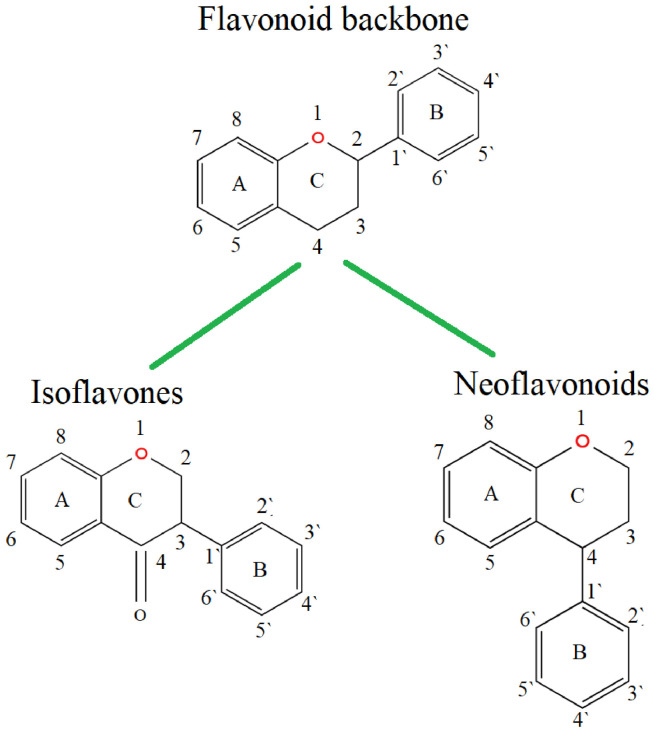
Basic skeleton structure of flavonoids, isoflavones, and neoflavonoids.

**Figure 2:**
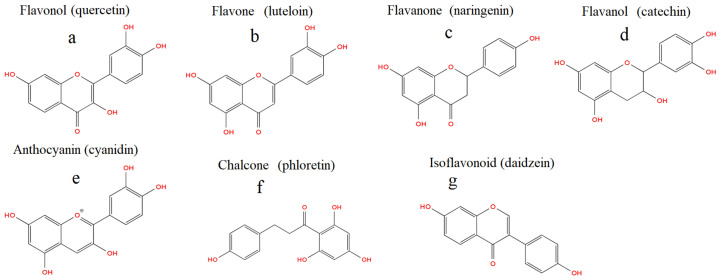
Chemical structures of some common representative flavonoids: (**a**) quercetin, (**b**) luteolin, (**c**) naringenin, (**d**) catechin, (**e**) cyanidin, (**f**) phloretin, (**g**) daidzein.

Flavonoids are classified into several subclasses based on the position at which the B ring attaches to the C ring, as well as the level of unsaturation and oxidation within the C ring (Figure 1). When the B ring is attached to the third carbon of the C ring, the compounds are known as isoflavones (Figure 1). If the B ring is connected at the fourth carbon, they are referred to as neoflavonoids. In contrast, flavonoids with the B ring linked at the second carbon of the C ring are further categorised into multiple subclasses according to variations in the structural characteristics of the C ring. These subclasses include flavonols, flavones, flavanonols, flavanones, anthocyanins, catechins, and chalcones (Figure 2).

### 2.1. Flavonols

Flavonols are a class of flavonoids that are built on a 3-hydroxyflavone scaffold, which is characterised by the presence of a ketone functional group. They are distinguished from other flavonoids by the presence of one or more phenolic hydroxyl groups at various positions on the scaffold. Quercetin, kaempferol, fisetin, and myricetin (Figure 2a) are among the most extensively studied flavonols. Compared with flavones, flavonols possess an additional hydroxyl group at the C-3 position of the C ring, which can also be glycosylated. Flavonols exhibit considerable structural diversity due to variations in hydroxylation and methylation patterns. When the wide range of possible glycosylated derivatives is considered, flavonols represent one of the most abundant and structurally diverse subclasses of flavonoids [22].

### 2.2. Flavones

Flavones constitute an important subgroup of flavonoids characterised by a double bond between positions 2 and 3 and a ketone group at position 4 of the C ring. Luteolin (Figure 2b) and apigenin are two common flavones. Most flavones possess a hydroxyl group at position 5 of the A ring, while additional hydroxylation commonly occurs at position 7 of the A ring or at the 3′ and 4′ positions of the B ring. These variations in hydroxylation contribute to the structural diversity and biological activities of flavones.

### 2.3. Flavanones

Flavanones represent another important subclass of flavonoids. Examples of flavanones include naringenin (Figure 2c), hesperitin, and eriodictyol. These compounds, also referred to as dihydroflavones, are characterised by a saturated C ring. In contrast to flavones, the double bond between positions 2 and 3 in the C ring is absent, which represents the main structural difference between these two flavonoid subgroups.

### 2.4. Flavanols

Flavanols, also known as dihydroflavonols or catechins (Figure 2d), are the 3-hydroxy derivatives of flavanones and represent a structurally diverse and highly substituted subgroup of flavonoids. Flavanols, commonly referred to as flavan-3-ols, contain a hydroxyl group attached to position 3 of the C ring. In contrast to many other flavonoids, these compounds lack a double bond between positions 2 and 3, which distinguishes their structural configuration.

### 2.5. Anthocyanins

Anthocyanins are glycosylated polyphenolic compounds that function as water-soluble pigments located in plant vacuoles. They produce a wide spectrum of colours ranging from orange and red to purple and blue, depending on the pH of the surrounding cellular environment in fruits, flowers, seeds, and other plant tissues [23]. Variations in the number and position of methoxy and hydroxyl groups attached to the flavylium core structure give rise to different anthocyanin molecules. To date, more than 650 anthocyanins have been identified in plants [24]. These compounds are generally classified into five principal groups: delphinidin, cyanidin (Figure 2e), pelargonidin, malvidin, and peonidin, along with their various derivatives [25].

### 2.6. Chalcones

Chalcones are naturally occurring open-chain flavonoids that may contain up to three modified or unmodified C5, C10, and C15 prenyl groups attached to their A and B aromatic rings. Major examples of chalcones include arbutin, phloridzin, chalconaringenin, and phloretin (Figure 2f). They serve as key biosynthetic precursors for isoflavonoids and flavonoids. Structurally, chalcones can be readily synthesised from simple aromatic compounds. They exhibit promising biological activities, which have driven the development of numerous chalcone analogues, as well as minor structural modifications of naturally occurring chalcones, resulting in a wide range of biologically active derivatives [26].

In flavonoids, the number and position of hydroxyl (-OH) groups play a critical role in determining biological activity. Hydroxyl substituents are typically attached to the benzene rings at positions such as 3, 5, 7, and 3′, 4′, and these substitution patterns strongly influence flavonoids’ bioactivity. The location of these -OH groups is also important; flavonoids containing a 3′, 4′-dihydroxy substitution pattern on the B ring and/or a hydroxyl group at the C-3 position generally exhibit strong radical scavenging activity [27]. Chalcones often contain one or more phenolic hydroxyl groups, which confer free radical scavenging activity and help counteract oxidative stress. Since oxidative stress is closely linked to inflammatory processes, its reduction may contribute to the suppression of inflammatory responses [26].

### 2.7. Isoflavonoids

Among the various classes of isoflavonoids, isoflavones are the most extensively studied and have attracted considerable attention because of their diverse biological activities. Isoflavones are characterised by the attachment of the B ring at the C-3 position of the heterocyclic C ring within the diphenylpropane (C6-C3-C6) backbone, which distinguishes them structurally from other classes of flavonoids. Isoflavones are generally categorised into three main groups: daidzein (Figure 2g), genistein, and glycitein [28].

## 3. Antimicrobial Activity of Flavonoids Against Gastrointestinal Pathogens

### 3.1. Chalcones Antimicrobial Activity Against Gastrointestinal Pathogens

**Figure 3:**
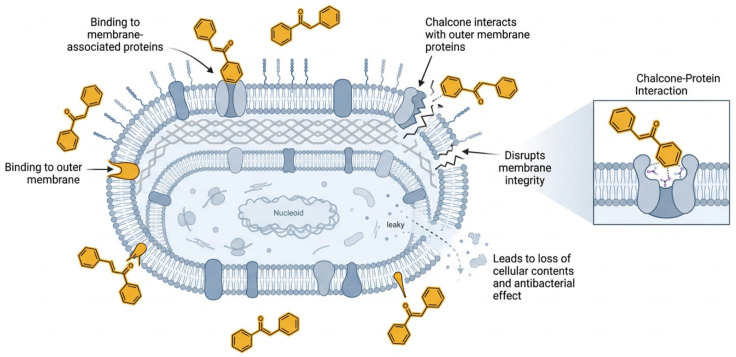
Chalcones exert an antimicrobial effect by disrupting bacterial cell wall integrity through binding to membrane-associated proteins and the outer membrane and interacting with outer membrane proteins, which disrupts membrane integrity and leads to the loss of cellular contents.

Chalcones isolated from *Treculia obovoidea* N.E.Br., including 4,2′,4′-trihydroxychalcone and its prenylated derivative, have notable antimicrobial activity against enteric pathogens, effectively inhibiting the growth of *Shigella flexneri*, *Proteus vulgaris*, *Citrobacter freundii*, *Salmonella typhimurium*, and *Enterobacter cloacae* (MICs in the range of 4.88–78.12 µg/mL) [29]. Similarly, prenylated chalcones kanzonol C (MIC = 4.9–39.1 µg/mL; 12.4–100 µM) and isobavachalcone (MIC = 0.3–39.1 µg/mL; 0.92–120 µM) were isolated from the stems of *Dorstenia barteri* Bureau. exhibited antimicrobial activity against all tested strains, including *S. typhimurium* [30]. The flavonoids colucins A and B isolated from *Colutea armata* Hemsl. & Lace. has also been reported to exhibit significant antimicrobial activity against *Pseudomonas aeruginosa*, *Escherichia coli*, *Salmonella typhi*, and *Burkholderia pseudomallei* (ZOI = 9–17 mm) [31]. Notably, MIC values were not determined in that study, making comparisons with other studies difficult. Isobavachalcone isolated from several species of the genus *Artocarpus* exhibited antimicrobial activity against *Pseudomonas putida* and *E. coli* (MIC = 450 µg/mL; 13,987 µM) [32]. Similarly, the hydroxylated chalcone, 2′,4′-dihydroxychalcone, exhibited good antimicrobial activity against *P. aeruginosa* and *E. coli* (MIC = 250 µg/mL; 1040 µM) [33]. However, the activity of 2′,4′-dihydroxychalcone was low compared with the positive controls, gentamicin (MIC = 4 µg/mL; 8.38 µM against *P. aeruginosa* and *E. coli*) and erythromycin (MIC = 62 µg/mL; 85 µM against *P. aeruginosa* and 125 µg/mL; 170 µM against *E. coli*) [33]. Similarly, the prenylated chalcone flemichin D also exhibited good antimicrobial activity against *P. aeruginosa* and *E. coli* (MIC > 150 µg/mL; >355 µM) [34]. The antimicrobial activity of chalcones depends on their structural variations, particularly the presence of prenyl and hydroxyl groups [35]. Studies have indicated that the antibacterial activity of chalcones may be due to their interaction with the bacterial cell wall (Figure 3). They can disrupt cellular integrity by binding to membrane-associated proteins and outer membranes [36]. However, further studies are needed to further evaluate their effects and confirm this proposed mechanism.

### 3.2. Flavanones Antimicrobial Activity Against Gastrointestinal Pathogens

**Figure 4:**
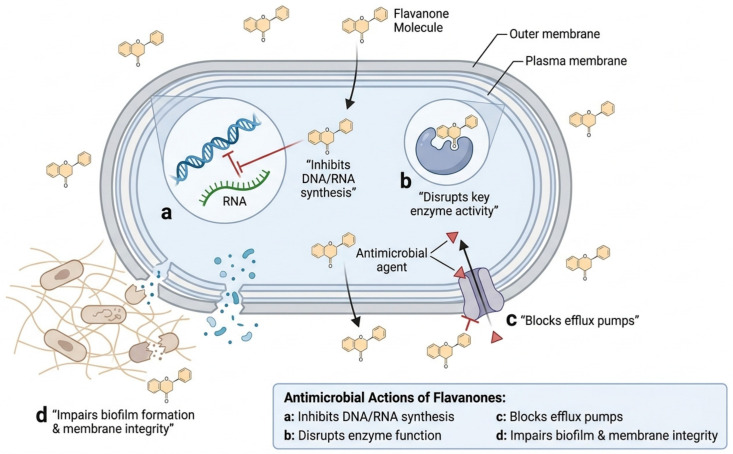
Flavanones exhibit antimicrobial effects by inhibiting the activity of (**a**) DNA, RNA, (**b**) enzymes, (**c**) efflux pumps, (**d**) the formation of biofilms and compromising membrane integrity.

Flavanones such as pinocembrin, naringenin, and 7-*O*-methyleriodictyol isolated from the species of the *Heliotropium* genus have significant antimicrobial activity against *E. coli*, *E. cloacae*, and *P. mirabilis* (MIC = 0.25–4 µg/mL) [37]. It has been suggested that the antimicrobial activity of these phytochemicals may be assisted by their physicochemical properties, such as diffusion coefficient and lipophilicity, and the presence of hydroxyl groups, which may aid penetration into the bacterial membrane. Nine prenylated flavonoids isolated from *Dorstenia mannii* Hook.f., including 6,8-diprenyleriodictyol and dorsamnins A-G, I, demonstrated strong antimicrobial activity against multidrug-resistant (MDR) strains of *E. aerogenes*, *E. coli*, and *P. aeruginosa* (MIC = 4–512 µg/mL) [38]. The prenylated flavanone 8,3′-diprenyl-5,7,4′-trihydroxyflavanone, isolated from *Flemingia strobilifera* (L.) W.T.Aiton, also exhibited strong antimicrobial activity against *E. coli* and *P. aeruginosa* (MIC = 17 µg/mL; 40 µM) [39]. Similarly, 6-prenyl pinocembrin isolated from *Pseudarthria hookeri* Wight & Arn. showed antimicrobial activity against *E. coli* and *P. aeruginosa* (MIC = 4–32 µg/mL; 15.6–125 µM) [40]. Studies have also recorded the antibacterial activity of prenylated flavanone lupinifolinol isolated from the roots of *Eriosema chinense* Vogel. against *P. aeruginosa* and *E. coli* (MIC > 150 µg/mL; >355 µM) [33]. The antibacterial activity was attributed to the presence of prenyl groups, although no detailed studies have examined the underlying mechanisms of action. Flavanone 5,7-dihydroxy-4′-methoxy-6-prenylflavanone isolated from *Artocarpus lowii* King and *Artocarpus anisophyllus* Miq. has moderate activity against *E. coli* (MIC = 900 µg/mL; 2539 µM; ZOI = 9 mm) [32]. Most of the flavanones tested in that study exhibited good antimicrobial activity against gastrointestinal pathogens, especially against *E. coli*. Suggested mechanisms of action involve suppressing nucleic acid synthesis, compromising membrane integrity, inhibiting efflux pumps or biofilm formation (Figure 4). However, many of these proposed mechanisms lack direct experimental validation, highlighting a significant gap in their mechanistic understanding. Integrated approaches such as electron microscopy, molecular biology studies, and molecular modelling are needed to confirm their mechanism of action. Furthermore, comprehensive toxicity studies should be conducted alongside antimicrobial studies, as many studies evaluate antimicrobial activity without determining cytotoxicity or therapeutic/safety indices, which are essential for confirming clinical safety. In addition, combination studies with conventional antibiotics are important to determine whether flavanones produce synergistic, additive, or antagonistic effects, thereby clarifying their potential role in enhancing efficacy, reducing resistance, or minimising adverse effects. Pharmaceutical formulation is also a critical step toward developing flavanones as therapeutic agents.

### 3.3. Antimicrobial Activity of Flavones Against Gastrointestinal Pathogens

Several studies have also emphasised the antimicrobial potential of flavones, particularly against gastrointestinal pathogens. Hispidulin, 6-methoxy-7-methyl-luteolin, apigenin-7-*O*-*β*-D-glucuronopyranoside, hispidulin-7-*O*-*β*-D-glucuronopyranoside, and apigenin-7-O-(6-methoxy)-*β*-D-glucuronopyranoside isolated from the *Centaurea pungens* Pomel., showed antimicrobial activity against *P. aeruginosa* (MIC = 100 µg/mL) [41]. Glycosylated flavonoids isolated from *Graptophyllum grandulosum* Turrill., including chrysoeriol-7-*O*-*β*-D-xyloside and its rhamnopyranosyl and apiofuranosyl derivatives, exhibited antimicrobial activity against multidrug-resistant *Vibrio cholerae* [MIC = 0.5–64 µg/mL]. The leakage of intracellular components, indicated by an increase in optical density at 260 nm, suggested that these compounds may act by destabilising bacterial membranes, ultimately leading to cell lysis [42]. Similarly, C-glycosyl flavones isolated from *Rhynchosia beddomei* Baker. including isovitexin and 5,7,3′,4′-tetrahydroxy-6-C-*β*-D-glucopyranosyl flavone, inhibited *E. coli* in agar diffusion assays (ZOI = 12.8 mm and 14.8 mm) and *P. aeruginosa* (ZOI = 19.1 mm and 17.5 mm) [43]. That study indicated that glycosidic moieties in flavonoids may enhance their antimicrobial activity. Additionally, molecular docking analysis revealed strong binding affinity to the *P. aeruginosa* peptide deformylase, suggesting a potential mechanism involving enzymatic inhibition.

Luteolin and chrysoeriol also displayed inhibitory activity against *P. aeruginosa* and *E. coli*, with MICs in the range of 1 µg/mL to 6.25 µg/mL [44]. Similarly, 5,7,4′-trimethoxyflavone isolated from *Praxelis clematidea* (Griseb.) R.M. King & H. Rob. also inhibited the growth of MDR *P. aeruginosa* and *E. coli* (MIC = 128 µg/mL; 409 µM) [45], whilst 3′,4′,7-trihydroxyflavone isolated from *Rhus verniciflua* Stokes substantially inhibited *E. coli* (MIC = 32 µg/mL; 118 µM) [46]. Isoflavones isolated from the acetone crude extracts of the stems of *Spatholobus parviflorus* (Roxb. Ex DC.) Kuntze inhibited the growth of *E. coli*, *S. typhimurium*, and *P. aeruginosa* (MIC = 32–128 µg/mL) [47]. The flavone 3,4′,5-trihydroxy-3′,7-dimethoxyflavone, isolated from *Dodonaea viscosa* subsp. *angustifolia* (L.f.) J.G.West, exhibits strong antibacterial activity against *E. coli*, with an MIC of less than 31.25 µg/mL (94.6 µM) [48]. Smilarly 5, 7-dihydroxy-4′-methoxyflavone, clerodendronone 1a, and clerodendronone 1b from *Clerodendrum formicarum* Gürke. inhibit the activity of *S. flexneri* (MIC = 62.5 µg/mL) [49]. Flavonoids isolated from *Pistacia integerrima* J.L.Stewart ex Brandis, including naringenin, 3,5,7,4′-tetrahydroxyflavanone, sakuranetin, and 3,5,4′-trihydroxy-7-methoxyflavanone, display antibacterial activity against *E. coli* (ZOI = 12–24 mm; MIC = 34–104 mg/mL) [50]. Vernoguinoflavone from *Vernonia guineensis* Benth. has moderate to low activity against *Salmonella muenchen* (*S. muenchen*), *E. coli*, and *S. typhimurium* [44]. Additionally, 5-carboxymethyl-4′,7-dihydroxyflavanone isolated from *Iris tenuifolia* Pall. showed inhibitory activity against *Helicobacter pylori* (*H. pylori*) and *E. coli* (MIC = 12.5–50 µg/mL) [51]. Flavones, especially those with methoxyl, hydroxyl, and glycosyl substitutions, show significant antimicrobial potential. They may function through membrane destabilisation or enzymatic inhibition, although further research is required to understand these mechanisms.

### 3.4. Flavonols Antimicrobial Activity Against Gastrointestinal Pathogens

Flavonols such as 3,3′-di-*O*-methylquercetin and 3-*O*-methylquercetin from *Dittrichia viscosa* (L.) Greuter inhibit the growth of *S. typhimurium* (MIC = 125 µg/mL). This effect is likely attributed to their capacity to compromise the integrity of the bacterial cell and viability through alterations in membrane permeability [52]. Flavonol, 3-*O*-methyl galangin, isolated from *Heliotropium* spp., exhibits strong antimicrobial activity against *E. cloacae* and *E. coli* (MIC = 0.25–1 µg/mL; 0.87–3.51 µM) [37]. Additionally, quercetin-3-*O*-*α*-L-rhamnopyranoside and kaempferol-3-*O*-*α*-L-rhamnopyranoside from *Albizia chinensis* (Osbeck) Merr. produced ZOIs of 16–17.5 mm against *E. coli* in agar diffusion assay models [53]. ZOIs demonstrate antimicrobial activity, although they do not provide a reliable measure of potency or allow accurate comparison between compounds. Therefore, MIC values are required for quantitative evaluation of antimicrobial efficacy. Despite this, quercetin and several of its glycosylated derivatives have demonstrated notable antimicrobial activity against *P. aeruginosa* and *E. coli*. Example includes quercetin-3′-O-*β*-D-glucopyranoside (ZOI = 21–26 mm), quercetin-7-O-methylether (ZOI = 16.8–20.4 mm), and quercetin-3-O-*α*-L-rhamnopyranoside-2″-gallate from *Salvia leucantha* Cav. (ZOI = 2–19 mm) [43,54,55]. However, as these findings are based on ZOIs, the antibacterial potency of these flavonols cannot be compared with other compounds or across studies. Quantitative antibacterial studies, particularly MIC evaluations, are required to validate the antimicrobial efficacy of quercetin and its glycosylated derivatives against *P. aeruginosa* and *E. coli*. Similarly, quercetin-3-*O*-*β*-D-glucosyl (1→4)-*α*-1-rhamnoside and quercetin show inhibitory activity against *S. typhimurium* (MIC = 3.12–50 µg/mL) [56]. Flavonols isolated from *Alternanthera maritima* (Mart.) A. St.-Hil., including kaempferol, quercetin 3-methyl ether, quercetin-3-*O*-rutinoside, and isorhamnetin-3-*O*-robinobioside, inhibit the growth of *E. coli*, *E. faecalis*, and *P. aeruginosa*, although the MIC reported in that study was high (MIC = 10–50 × 10^4^ µg/mL), suggesting differences in antimicrobial activity amongst structurally related compounds [57]. However, such a high MIC value raises questions about the validity of this study. Due to the relatively low solubility of flavonoids, it is unlikely that these concentrations could be achieved without the use of solvents, which may affect the apparent biological activity of these compounds. Therefore, these results need to be verified in future studies. However, if these results ultimately are confirmed, they may indicate that structural features such as methylation, hydroxylation, and glycosylation may influence the antimicrobial activity of these closely related flavonols. In general, glycosylation tends to reduce activity due to increased polarity and reduced membrane permeability, whereas methylation may enhance lipophilicity and improve interactions with bacterial targets.

Quercetin-5,3-dimethylether, rhamnocitrin, and rhamnazin exhibit MICs in the range of 25–100 µg/mL against *E. faecalis*, *Vibrio cholerae*, and *Shigella sonnei* [58]. Isorhamnetin 3-O-*β*-D-rutinoside and isorhamnetin 3-*O*-*β*-D-glucoside completely inhibited the growth of *E. coli* (MICs = 10–26 µM) [59]. Astragalin (kaempferol 3-*O-β*-D-glucoside) and kaempferol 3-*O*-[3-*O*-acetyl-6-*O*-(E)-p-coumaroyl]-*β*-D-glucopyranoside isolated from *Scabiosa hymettia* Boiss. & Spruner show antimicrobial activity against *E. coli*, *E. cloacae*, and *P. aeruginosa* (ZOI = 10–13 mm) [60]. However, the absence of quantitative potency measurements such as MIC values limits the interpretation of these findings. Similarly, the flavonols isorhamnetin-3-*O*-rutinoside, kaempferol-3-*O*-rutinoside, and quercetin-3-*O*-rutinoside isolated from *Calotropis procera* (Aiton) W.T.Aiton displayed MICs of 40–320 µg/mL and ZOI of 15.5– 28.5 mm against *Bacillus subtilis*, *P. aeruginosa*, and *S. enteritidis* [61]. It has been suggested that the antimicrobial activity of these compounds may be associated with their ability to form complexes with soluble and extracellular proteins, as well as bacterial metabolites such as phosphate and glutamate, which results in changes in membrane permeability and disruption of peptidoglycan structure [61]. The presence of hydroxyl groups may also play a role in enhancing their antimicrobial efficacy. However, additional studies are required to validate these proposed mechanisms. Previous studies have shown that sugar moieties and hydroxyl groups significantly influence the bioactivity of flavonols, possibly through interactions with cell walls and bacterial proteins [62]. In that study, quercetin-3-*O*-*β*-D-glucopyranoside demonstrated notable antibacterial activity (MIC = 32–64 µg/mL; 70 µM–138 µM), effectively eliminating *S. flexneri* and *V. cholerae* [62]. Moderate antimicrobial activity was also recorded for quercetin 3-*O*-*β*-rutinoside and isorhamnetin 3-*O*-*β*-rutinoside from *Galium brunneum* Munby against *B. cereus*, *P. aeruginosa*, and *E. coli* in agar diffusion assays (ZOI = 7–10.3 mm) [63]. However, the absence of MIC or other quantitative potency data limits the interpretation of these findings. The antimicrobial efficacy of these compounds should be further validated using a standardised susceptibility assay. The antimicrobial potential of quercetin has been demonstrated against several gastrointestinal pathogens, including *S. flexineri*, *E. coli*, *Salmonella muenchen* (*S. muenchen*), *S. typhi*, and *S. typhimurium* (MIC = 6.25–50 µg/mL; 21 µM–165 µM) [44].

Fustin and fisetin from *Toxicodendron vernicifluum* (Stokes) F.A. Barkley inhibit the growth of *E. coli*, with MICs of 8 and 63 µg/mL (27.75–4543 µM), respectively, but did not inhibit the growth of *S. typhimurium* [52], indicating a potentially selective mechanism of action. In studies screening against clinical bacterial strains, ericoside exhibited greater antibacterial activity against an MDR *E. coli* strain (MIC = 64 µg/mL; 74 µM), whilst showing comparatively moderate effects against standard strains (MIC = 128 µg/mL; 147 µM) [64]. The authors suggested that the antimicrobial activity may be associated with interactions with bacterial membranes. However, a more comprehensive investigation is required to fully elucidate the underlying mechanisms. Structural elements, including glycosides, hydroxyl groups, sulfation, and methylation, play a significant role in modulating the activity of flavonols. The proposed mechanism of action of flavonols includes the inhibition of essential enzymes, disruption of membrane integrity, and interaction with bacterial proteins [43]. Future studies should focus on in vivo validation, structure-activity relationships, formulation development, and toxicity evaluation to support the development of flavonols as effective antimicrobial agents.

### 3.5. Isoflavonoids Antimicrobial Activity Against Gastrointestinal Pathogens

Although the potency of isoflavonoids varies considerably, certain isoflavonoids have demonstrated promising antimicrobial activity against gastrointestinal pathogens. Isoflavone 7,4′-dihydroxy-5,3′-dimethoxyisoflavone isolated from *Hypericum oblongifolium* Choisy inhibits the growth of *E. coli*, *S. typhi*, and *P. aeruginosa* (ZOI = 17–23 mm) [54]. However, as these findings are based solely on ZOIs, they do not provide quantitative information on antimicrobial potency or allow for comparison with other compounds. Further evaluation using MIC is therefore necessary. Molecular docking studies suggest that the antimicrobial activity may be due to the inhibition of several enzymes, including GlcN-6-P synthase and 5-lipoxygenase (5-LOX). Genistein, a prenylated isoflavone isolated from *Flemingia strobilifera* (L.) W.T.Aiton, also demonstrated antimicrobial activity against *E. coli* (MIC = 146 µg/mL; 540 µM) and *P. aeruginosa* (MIC = 136 µg/mL; 503 µM) [39]. The prenylated isoflavones 6, 8-diprenylgenistein, abyssinone V 4′-*O*-methyl ether, burtinone, and alpinumisoflavone, extracted from *Erythrina caffra* Thunb., have notable antimicrobial activity against *E. coli* (MIC = 3.9–62 µg/mL), indicating that prenylation may facilitate membrane penetration and increase lipophilicity [65]. Similar isoflavones from *Spatholobus parviflorus* (Roxb.ex DC.) Kuntze and *Millettia extensa* (Benth.) Benth. ex Baker. also exhibit activity against *S. typhimurium* (MIC = 64–128 µg/mL) [47,66]. Derrone, a prenylated isoflavone isolated from the flowers of *Retama raetam* (Forssk.) Webb & Berthel., has significant antimicrobial activity against *P. aeruginosa* and *E. coli* (MIC = 7.81–15.62 µg/mL; 23.22–46.44 µM; ZOI = 19–16 mm) [67]. It has been suggested that derrone may exert antimicrobial effects by forming protein complexes and interacting with the bacterial cell wall. In contrast, lachnoisoflavone A and B, isolated from *Crotalaria lachnophora* A.Rich. exhibited moderate antimicrobial activity against *E. coli* in agar diffusion assays (ZOI = 7–8.3 mm) [68]. To establish the therapeutic relevance of lachnoisoflavones, quantitative susceptibility studies such as MIC are required. Isoflavonoids exhibit significant antimicrobial activity, and further analysis is needed for their advancement towards clinical application. Studies should focus on toxicity, pharmacokinetic properties, stability, and the mechanisms of action. Clinical trials and in vivo studies are also required to validate their use as an alternative antibacterial agent.

### 3.6. Flavanols Antimicrobial Activity Against Gastrointestinal Pathogens

Flavanols have antimicrobial activity against gastrointestinal pathogens, although their effectiveness varies substantially. The flavanol, epiafzelechin, isolated from *Ficus cordata* Thunb. has antimicrobial activity against a wide range of gastrointestinal pathogens, including *S. dysenteriae*, *E. coli*, and *S. typhi* (MIC = 19.53–78.12 µg/mL; 71.20–284 µM) [69]. Flavanols, including catechin and epicatechin extracted from *Schotia latifolia* Jacq., also inhibited the activity of *E. coli* and *P. aeruginosa* (MIC = 62.5–125 µg/mL; 215–430 µM) [70]. Epicatechin and a flavan derivative from *Embelia schimperi* Vatke moderately inhibited the growth of *S. dysenteriae* and *E. coli* (ZOI = 6–12 mm for epicatechin and ZOI = 10–13 mm for the flavan derivative) [71]. Without quantitative susceptibility data such as MIC values, it is not possible to compare the efficacy of these compounds. Studies have also reported the antimicrobial activity of epicatechin isolated from *Senegalia polyacantha* (Wild.) Seigler & Ebinger and *Maytenus buchananii* (Loes.) R.Wilczek against *S. flexneri*, *V. cholerae*, and *P. aeruginosa* (MIC = 32–256 µg/mL; 110–881 µM) [62,72]. It has been suggested that the main antimicrobial mechanism of flavanols includes changing the permeability of the bacterial membrane, disrupting critical metabolic pathways, or inhibiting key enzymes. However, future studies need to clearly identify and verify the antibacterial mechanisms of flavanols. Studies should also evaluate their in vivo stability, molecular targets, bioavailability, toxicology, and pharmacodynamic studies to harness the therapeutic potential of these compounds.

### 3.7. Flavanonol Antimicrobial Activity Against Gastrointestinal Pathogens

The ethylated flavanonol, 3-*O*-ethyl-dihydroquercetin, extracted from *Manilkara hexandra* (Roxb.) Dubard exhibited potent antimicrobial activity against *E. coli*, *S. typhimurium*, and *P. aeruginosa* in agar diffusion (ZOI = 20.3–22.9 mm) and quantitative assays (MIC = 0.98–3.9 µg/mL; 2.94–11.7 µM) [73]. However, the study did not confirm that structural modifications, including ethylation, contribute to the observed antimicrobial activity. Dihydroflavonol isolated from *Tamarix nilotica* (Ehrenb.) Bunge shows inhibitory activity against *S. typhi* and *E. coli* in agar diffusion assays (ZOI = 16–14 mm) [74]. However, as these findings are based solely on ZOIs, the antimicrobial potency of this compound cannot be determined. Quantitative studies, such as MIC evaluations, are required to validate its antimicrobial efficacy and allow comparisons with other compounds. Additionally, glycosylated flavanonol taxifolin 3-*O*-*α*-L-rhamnopyranoside from *Erica mannii* (Hook.f.) Beentje moderately inhibit the activity of *E. aerogenes* and *E. coli* (MIC = 128 µg/mL; 284 µM) [64]. Tetraflavonoids, lemairone A and B from the leaves of *Zanthoxylum lemairei* (De Wild.) P.G.Waterman, demonstrates weak to moderate activity against *E. coli* (MIC = 128 µg/mL; 113 µM) for lemairone A and MIC = 64 µg/mL (58 µM) for lemairone B [75]. The observed difference in the antimicrobial activity of lemairone A and B suggests that any minor structural variations among tetraflavonoids can influence their antibacterial activity substantially. Nevertheless, significant gaps exist in understanding the antimicrobial mechanism of flavanonols and tetraflavonoids. Similarly, a lack of data regarding stability and toxicity also limits their progression towards clinical applications.

### 3.8. Anthocyanins Antimicrobial Activity Against Gastrointestinal Pathogens

Studies have shown that an anthocyanin extract from crude pomegranate juice (20 mg per disc) inhibited the growth of *E. coli* (ZOI = 13 mm) [76]. However, a purified juice pomegranate extract (4 mg per disc), which is an aliquot of the crude extract purified by solid phase extraction, showed a ZOI of 8 mm against *E. coli*. The study has investigated the antimicrobial activity of anthocyanins of crude and purified pomegranate juice against *E. coli* at two different concentrations, 20 mg and 4 mg per disc, which may explain the observed differences in ZOI. Alternatively, it might be possible that in the crude extracts there are other phytochemicals present that are synergising the antimicrobial activity of anthocyanins and producing a high ZOI against *E. coli*. However, the study should have used the same concentration in both the crude and purified extracts to facilitate easier comparison. In the dilution tube method, pomegranate juice extract also inhibited the activity of *E. coli* but at a very high concentration (MIC = 40 µg/µL) [76]. Ampicillin, a positive control used in the study, produced a higher ZOI (30 mm) against *E. coli* at a much lower concentration (80 µg per disc) [76]. Anthocyanins extracted from *Hibiscus sabdariffa* L. showed antimicrobial activity against *E. coli* at 25 mg/mL (ZOI = 13.63 ± 1 mm) [77]. However, the study did not report the MIC values to allow comparison with other compounds. Anthocyanins extracted from Turkish *Cinclidotus fontinaloides* (Hedw.) P.Beauv. restrict the growth of *E. coli* at 10 mg/mL [78]. Similarly, anthocyanins extracted from *Withania somnifera* (L.) Dunal also inhibited the growth of *E. coli* (ZOI = 15 mm; MIC = 25 mg/mL) [79]. Most of these studies have investigated the antimicrobial activity of anthocyanins using preliminary antimicrobial assays, including disc diffusion assay and liquid dilution assay, without providing any insight into their mechanism of action. Future studies should look into the antimicrobial mechanism of anthocyanins to better understand their mode of action and therapeutic potential against gastrointestinal pathogens.

The antimicrobial potential of anthocyanins has also been studied against other gastrointestinal pathogens, including *Salmonella* and *S. typhimurium*. Anthocyanins extracted from pomegranate have demonstrated antimicrobial activity against *Salmonella* species, with reported MIC values ranging from 10.75 to 12.5 mg/mL [80]. Anthocyanins extracted from lowbush blueberries exhibited antimicrobial activity against *S. typhimurium* (MIC = 34.75 mg/mL) [81]. Anthocyanins extracted from 21 different European cranberry berry samples inhibited the growth of *S. typhimurium*, with ZOIs ranging from 13.33 ± 0.47 to 22.33 ± 1.41 mm [82]. Anthocyanins extracted from different clones of *Viburnum opulus* L. fruit juice inhibited the growth of *S. typhimurium*, with the ZOIs value ranging from 25.3 ± 0.42 to 30.3 ± 0.47 mm [83]. In another study, anthocyanins extracted from *Vaccinium floribundum* Kunth produced 20 mm ZOI against *S. typhimurium*, which was closer to the positive ampicillin (ZOI = 23 mm) [84]. Anthocyanins have also been extracted from red cabbage and inhibited the activity of *S. typhimurium* (MIC = 400 mg/mL; MBC = 500 mg/mL) [85]. Most of these studies have investigated the antimicrobial activity of anthocyanins extracted from the natural product using a disc diffusion assay. Future studies should evaluate the antimicrobial activity of anthocyanins using quantitative assays such as the broth microdilution assay to measure the MIC. This will allow us to compare the antimicrobial efficacy of anthocyanins to other compounds.

## 4. Flavonoids Antimicrobial Mechanisms

Flavonoids often display varying levels of activity against different bacterial species. The mechanism of action of flavonoids can be multifactorial and complex [86]. Some of the mechanisms include inhibiting efflux pumps, changing the integrity of the membrane and cell wall, and inhibition of energy metabolism, nucleic acid synthesis, cell proliferation, and protein synthesis. Flavonoids can also reduce cellular adhesion, biofilm formation, attenuate pathogenicity, inhibit intracellular enzymes, and induce oxidative stress [70]. The presence of an outer membrane rich in lipopolysaccharides makes the development of effective antimicrobial agents against Gram-negative bacteria particularly challenging [86]. However, some studies have indicated that the antimicrobial effects of flavonoids may result from their interactions with the bacterial cell membrane. This membrane plays an important role in protecting the cell, acting as a selective barrier that controls the movement of substances whilst also maintaining the proper function and integrity of the cell structure [87]. In addition to the peptidoglycan layer and cytoplasmic inner membrane, the presence of an outer membrane also hinders the entrance of antimicrobial agents into the cell and increases bacterial antibiotic-resistance [37]. Alterations to the bacterial membrane induced by the antimicrobial agents can result in disruptions to cellular homeostasis and metabolic dysfunction and may ultimately cause membrane rupture, resulting in bacterial death [88]. However, studies investigating the molecular mechanisms underlying flavonoid-membrane interactions remain limited, and the exact mode of action is not yet fully understood [89].

**Figure 5:**
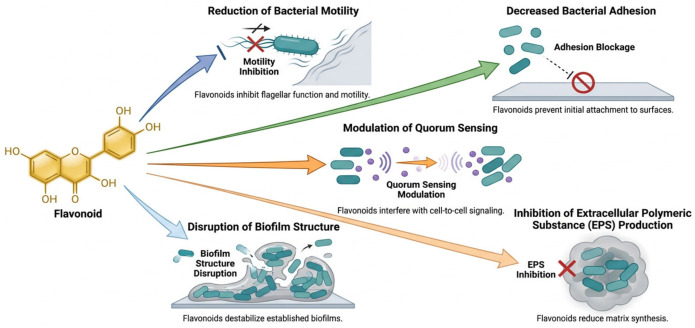
Flavonoids inhibit the formation of bacterial biofilm through different mechanisms, including reduction in bacterial motility, decreased bacterial adhesion, modulation of quorum sensing, disruption of biofilm structure, and inhibition of the production of extracellular polymeric substances.

The antimicrobial activity of flavonoids may depend on their structural properties, enabling them to interact differently with bacterial targets, including enzymes, efflux pumps, and bacterial cell membranes [90] (summarised in Figure 5). However, more comprehensive studies are required to elucidate how the chemical structure of flavonoids affects their interactions with these complex bacterial targets. Bacteria diminish the efficacy of antimicrobial agents by expressing efflux pumps, which actively transport antibiotics out of the cell [91]. Efflux pumps play a significant role in the survival of bacterial cells by stabilising biofilm and protecting against oxidative stress. Biofilms play a key role in facilitating cell adhesion to surfaces and providing protection against harsh environmental conditions, including immune responses, dehydration, and antimicrobial agents. Studies have shown that flavonoids can suppress the expression of genes linked to bacterial virulence and biofilm formation. They act on multiple targets through various mechanisms, including reduction in bacterial motility and adhesion, modulation of quorum sensing, disruption of biofilm structure and inhibition of extracellular polymeric substance (Figure 5) [92,93].

## 5. Flavonoids and Antibiotic Synergistic Interactions Against Gastrointestinal Pathogens

Synergistic interactions involve combining two or more components to determine whether their combined effect is significantly greater than the sum of their individual effects [94]. According to the World Health Organisation (WHO), combinational therapies are preferred over monotherapies for life-threatening infectious diseases because they may target multiple aspects of the diseases and help prevent the development of bacterial resistance [95]. The ability of plant-derived compounds to enhance or restore the effectiveness of conventional antibiotics in treating microbial infections may have a significant impact on global health by helping to combat resistant pathogenic microorganisms. Microorganisms have evolved several mechanisms to resist antibiotics, with one of the most common being the use of MDR efflux pumps. These efflux pumps are often chromosomally encoded and function by actively expelling antibiotics that enter bacterial cells, thereby reducing intracellular drug accumulation and contributing to antibiotic resistance [96]. A single efflux pump can enable bacteria to resist multiple classes of antimicrobial agents. Inhibition of these efflux pumps increases the intracellular concentrations of antibiotics, thereby restoring their therapeutic effectiveness. Interestingly, flavonoids can act as MDR pump inhibitors, enhancing the activity of antibiotics in combination therapies. Such MDR pump inhibitors can serve as valuable tools when used in combination with previously ineffective or resistance-prone antibiotics. For example, orientin in combination with gentamicin or erythromycin produced synergistic interactions against *S. sonnei* by blocking efflux pumps [97]. Flavonoid–antibiotic combinations not only enhance the overall antimicrobial effect but can also function as resistance-modifying agents. Several studies have recorded the synergistic interactions between flavonoids and conventional antibiotics against gastrointestinal pathogens (summarised in Table 1).

## 6. Challenges and Future Direction

Plant-derived flavonoids have demonstrated potential in inhibiting the activity of gastrointestinal pathogens. However, most studies to date have primarily focused on assessing the presence or absence of antibacterial activity, with limited exploration of their mechanisms of action, toxicity profiles, or clinical profiles. Many of those studies have focused on measuring ZOIs or MICs against various gastrointestinal pathogens, without investigating other important aspects, such as distinguishing between bacteriostatic and bactericidal effects, or interactions between conventional antibiotics and flavonoids. Whilst these preliminary studies are valuable for demonstrating antimicrobial activity, a deeper understanding of the underlying mechanism of action is essential. More comprehensive investigations are needed to evaluate the toxicity and pharmacokinetics of these flavonoids, particularly through advanced clinical studies and animal models. Given that most studies have been conducted in vitro, it is important to examine the antimicrobial efficacy of flavonoids in vivo to better understand their overall safety and effects. Furthermore, molecular docking analysis would also aid in determining their mechanism of action. Expanding on these investigations, along with elucidating how flavonoids inhibit essential enzymes, interact with bacterial membranes, and interfere with the biosynthesis of key cellular components, may yield valuable insights into their efficacy. Such mechanisms highlight their potential use as adjuvants and may lead to the development of more effective, novel, and targeted antimicrobial agents to help combat the growing problem of gastrointestinal infections.

Flavonoids have been reported to favourably modulate the gut microbiota by increasing beneficial bacterial populations and reducing potentially harmful microorganisms [108]. However, studies specifically evaluating their selectivity toward gastrointestinal pathogens relative to commensal gut bacteria remain limited. Future studies should investigate the activity of flavonoids against a wider panel of gastrointestinal pathogens. Given that certain flavonoids can enhance bacterial susceptibility and even reverse antimicrobial resistance. It is important to assess their effects in combination with other compounds. Combining flavonoids with antibiotics that act through different mechanisms against gastrointestinal pathogens can enhance therapeutic efficacy, promote a synergistic effect, and potentially allow for reduced dosages of both agents. Additionally, the development of advanced delivery systems, such as flavonoid-based nanoparticles, should be considered as a strategy to improve bioavailability, targeted delivery, and stability of these compounds at the site of infection.

## 7. Conclusions

Several studies have demonstrated the antimicrobial efficacy of flavonoids against gastrointestinal pathogens. However, despite these advances, there are still significant gaps in understanding their efficacy and potential application in the development of new therapies to combat AMR. A more comprehensive understanding of their mechanisms of action, properties, and interactions with other molecules is essential for advancing the development of new antimicrobial therapies. Future studies should also prioritise in vivo studies to validate the efficacy of flavonoids under physiological conditions, as well as investigate flavonoid–antibiotic interactions to achieve synergistic effects. Such approaches are important for assessing the therapeutic potential of flavonoids against gastrointestinal pathogens.

## Figures and Tables

**Table 1:** Flavonoid/antibiotic synergistic interactions against GI pathogens.

Synergistic Combination	Against Bacteria	∑FIC Values	Reference
Gentamicin and epigallocatechin-3-gallate	*E. coli*	0.5	[98]
Gentamicin and luteolin	*E. coli*	0.31	[98]
Levofloxacin and epigallocatechin-3-gallate	*E. coli*	0.28	[98]
Levofloxacin and luteolin	*E. coli*	0.37	[98]
Ampicillin and epigallocatechin-3-gallate	*E. coli*	0.25	[98]
Ampicillin and luteolin	*E. coli*	0.5	[98]
Trimethoprim and luteolin	*E. coli*	0.31	[98]
Amikacin and naringenin	*E. coli*	0.31	[99]
Colistin and baicalin	*E. coli*	0.25	[100]
Amoxicillin and luteolin	*E. coli*	0.12	[101]
Epigallocatechin gallate and gentamicin	MDR *E. coli*	0.32	[102]
Epigallocatechin gallate and cefotaxime	ESBL *E. coli*	Not reported	[103]
Galangin and amoxicillin	Amoxicillin-resistant *E. coli*	<0.09	[103]
Kaempferide and amoxicillin	Amoxicillin-resistant *E. coli*	<0.09	[104]
Kaempferide-3-O-β-D-glucoside and amoxicillin	Amoxicillin-resistant *E. coli*	<0.09	[104]
Gentamicin and apigenin	*P. aeruginosa*	0.37	[98]
Gentamicin and luteolin	*P. aeruginosa*	0.37	[98]
Levofloxacin and apigenin	*P. aeruginosa*	0.37	[98]
Levofloxacin and epigallocatechin-3-gallate	*P. aeruginosa*	0.5	[98]
Levofloxacin and myricetin	*P. aeruginosa*	0.12	[98]
Tobramycin and quercetin	*P. aeruginosa*	0.25	[105]
Amikacin and quercetin	*P. aeruginosa*	0.50	[105]
Ceftriaxone and quercetin	*P. aeruginosa*	0.37	[105]
Gentamicin and quercetin	*P. aeruginosa*	0.50	[105]
Levofloxacin and quercetin	*P. aeruginosa*	0.50	[105]
Colistin and kaempferol	*P. aeruginosa*	0.07	[106]
Epigallocatechin gallate and tetracycline	*Vibrio cholerae*	0.009	[107]
Orientin and gentamicin	*S. sonnei*	0.50	[97]
Orientin and erythromycin	*S. sonnei*	0.25	[97]

## Data Availability

No new data were created or analyzed in this study. Data sharing is not applicable to this article.
